# Engineering of *Sulfolobus acidocaldarius* for Hemicellulosic Biomass Utilization

**DOI:** 10.4014/jmb.2202.02016

**Published:** 2022-02-24

**Authors:** Areum Lee, Hyeju Jin, Jaeho Cha

**Affiliations:** 1 Department of Integrated Biological Science, College of Natural Sciences, Pusan National University, Busan 46241, Republic of Korea; 2 Department of Microbiology, College of Natural Sciences, Pusan National University, Busan 46241, Republic of Korea; 3 Microbiological Resource Research Institute, Pusan National University, Busan 46241, Republic of Korea

**Keywords:** Hyperthermophiles, *Sulfolobus acidocaldarius*, hemicellulose, carbohydrateactive enzyme

## Abstract

The saccharification of cellulose and hemicellulose is essential for utilizing lignocellulosic biomass as a biofuel. While cellulose is composed of glucose only, hemicelluloses are composed of diverse sugars such as xylose, arabinose, glucose, and galactose. *Sulfolobus acidocaldarius* is a good potential candidate for biofuel production using hemicellulose as this archaeon simultaneously utilizes various sugars. However, *S. acidocaldarius* has to be manipulated because the enzyme that breaks down hemicellulose is not present in this species. Here, we engineered *S. acidocaldarius* to utilize xylan as a carbon source by introducing xylanase and β-xylosidase. Heterologous expression of β-xylosidase enhanced the organism’s degradability and utilization of xylooligosaccharides (XOS), but the mutant still failed to grow when xylan was provided as a carbon source. *S. acidocaldarius* exhibited the ability to degrade xylan into XOS when xylanase was introduced, but no further degradation proceeded after this sole reaction. Following cell growth and enzyme reaction, *S. acidocaldarius* successfully utilized xylan in the synergy between xylanase and β-xylosidase.

## Introduction

Today, many chemical production and energy system industries rely on fossil fuels, which are also a major factor in global warming and air pollution [1, 2]. According to the Intergovernmental Panel on Climate Change (IPCC), more than 90% of global warming since the mid-20th century is due to the increase in the concentration of greenhouse gases such as CO_2_, and fossil fuels are the main cause behind the artificial emission of CO_2_ [3, 4]. Many countries worldwide are trying to reduce and limit the use of fossil fuels to lower the concentration of greenhouse gases in the atmosphere. Governments, including those of European Union member states, are striving to achieve net-zero carbon without negatively affecting economies during the second half of this century [5]. Moreover, the withdrawal of fossil fuel investment, a social movement, that calls for the abolition of financial investment in fossil fuels, has rapidly spread around the world in the last decade [6, 7]. Therefore, renewable biomass as an alternative to fossil fuels is attracting attention.

Lignocellulosic biomass (LB), or plant dry matter, is considered a second-generation biofuel producer. LB comprises various materials, including agricultural wastes, forest residues, and short rotifers and crops [8]. Since LB is one of the most abundant raw materials on Earth, biofuels made from LB have economic advantages. In the late 1970s, the US Department of Energy launched a program to convert LB into ethanol and the National Renewable Energy Laboratory later researched biomass conversion to ethanol [9].

LB is mainly composed of cellulose (40–50%), hemicelluloses (25–30%), and lignin (15–25%) [10]. While cellulose is a polymer composed of glucose with β-1,4 linkages, hemicellulose composition varies depending on the type of biomass. Hemicelluloses are composed of hexoses (glucose, galactose, and mannose), pentoses (xylose and arabinose), and uronic acids (glucuronic, galacturonic, and methylgalacturonic acid), and can be classified into xylans, xyloglucans, mannans, and mixed-linkage glucans. A previous study by Kumar *et al*. found that xylose was most abundant in hemicellulose [11, 12]. Xylan, the major hemicellulose in the primary cell wall of commelinid monocots, mainly consists of a xylose backbone with β-1,4 linkages, and other compounds, such as galactose, arabinose, ferulic acid, and glucuronic acid, are linked to the backbone [10, 13]. To convert LB into biofuel, the decomposition of these complex, highly polymeric compounds into simple sugars must precede.

*Sulfolobus* could be a good candidate for saccharification as this genus harbors various well-known sugar transport and degradation pathways [14, 15]. *Sulfolobus* can utilize hexose and pentose sugars simultaneously because it does not have catabolite repression [16]. As this genus grows at high temperatures and acidic conditions, using *Sulfolobus* for saccharification may provide additional benefits, such as the reduced risk of contamination and increased substrate solubility [15]. Among *Sulfolobus* species, *Sulfolobus acidocaldarius*, a thermoacidophilic archaeon isolated from geysers in Yellowstone National Park by Brock *et al*. [17], has proved to be a good candidate for hemicellulose saccharification. To engineer the microbial metabolic pathways for efficient decomposition of hemicellulose, *S. acidocaldarius* offers the advantage of being genetically stable with a well-developed genetic manipulation system without a mobile element. Some of the other *Sulfolobus* genomes harbor active IS elements, which can lead to genetic instability [18, 19]. *S. acidocaldarius* was previously identified to transport pentose sugars, such as xylose and arabinose, using ABC transporters, and to utilize these sugars via the Weimberg and Dahms pathways [20, 21]. However, *S. acidocaldarius* cannot saccharify xylan, whereas *Saccharolobus solfataricus*, a neighboring strain of *S. acidocaldarius*, can degrade xylan into xylose using xylanase and β-xylosidase [22, 23].

In this study, we introduced xylanase and β-xylosidase to develop *S. acidocaldarius* strains for hemicellulose saccharification, especially the hemicellulose with xylan. The genes, *sso1354* and *sso3032*, encoding xylanase and β-xylosidase, respectively, were amplified from *S. solfataricus* P2 and introduced into *S. acidocaldarius* by ligation into chromosomal DNA and plasmid transformation [23, 24]. Xylan utilization of the constructed strain was confirmed by growth analysis and enzyme activity assay.

## Materials and Methods

### Chemicals and Reagents

Xylose, glucose, and xylooligosaccharide (XOS) were purchased from Sigma-Aldrich (USA). Xylan extracted from beechwood was purchased from Megazyme, Inc. (Ireland), and carboxymethyl cellulose (CMC) was from Calbiochem (USA). The Bio-Rad protein assay dye reagent used for Bradford assay was from Bio-Rad (USA). Other artificial carbohydrates, pNPX and RBB-xylan, were purchased from Sigma. PRIME STAR polymerase from Takara Bio, Inc. (Japan) and n*Taq* polymerase from Enzynomics (Daejeon, Korea) were used for DNA amplification. For restriction and ligation, *Sap*I was purchased from NEB (USA), and the other restriction enzymes and T4 DNA ligase were purchased from Takara. Silica gel 60 F254 thin layer chromatography (TLC) plate was purchased from Merck (Germany). Other chemicals were purchased from Junsei (Japan), Daejung (Korea), and Bioshop (Canada).

### Growth Conditions

*S. acidocaldarius* strains were grown in Brock’s basal media supplemented with 0.2% NZ-amine at 77°C [17]. Various sugars, including xylose, glucose, XOS, xylan, cellobiose, and CMC were provided as carbon sources and were added with a final concentration of 0.2%. Moreover, 0.02% dextrin was supplemented to the media to induce the expression of β-xylosidase. Finally, for the growth of uracil-auxotroph strains, 20 mg/L of uracil was supplemented.

### Mutant Construction

Strains and primers used in this study are listed in Tables 1 and 2. *S. acidocaldarius* MW001, a uracil-auxotroph, due to the deletion of 322 bp on the orotate phosphoribosyltransferase (*pyrE*) gene [18], served as a wild-type strain. Mutant construction was conducted by two methods: plasmid transformation and markerless insertion of the gene. To construct vectors for gene expression, *sso3032* and *sso1354* genes were amplified and cloned into pSVAmalFX-Nt6H and pC, respectively. For amplification, the genomic DNA of *S. solfataricus* P2 was used as a template. The amplified *sso3032* was ligated into pSVAmalFX-NtH6 by *Sap*I restriction and ligation [25]. For *sso1354* expression in the pC vector, *sso1354* was fused with the promoter (455 bp) and terminator regions (500 bp) of *gdhA* and further ligated into the pC vector, resulting in the construction of pC::*sso1354*. To avoid SuaI restriction, the constructed plasmids were methylated by transformation to *Escherichia coli* ER1821 [26, 27]. The transformation of MW001 with the methylated vector was conducted by electroporation (1.5 kV, 25 μF, 1,000 Ω, 1 mm). Thus, the mutants harboring β-xylosidase and xylanase were named MW001/3032 and MW001/1354, respectively.

For the construction of the *LAR1* strain, markerless insertion of the *sso3032* into the *pyrE*–*pyrF* region of *S. acidocaldarius* was conducted using the method by Wagner *et al*. with modification [18]. First, 390 bp of *pyrE* (*saci_1597*) and *pyrF* (*saci_1598*) were amplified and named after U and D to exchange partial *pyrE*–*pyrF* gene into *sso3032*. Then, the U–D fragment, made by fusion PCR using U and D fragment, was cloned to the Tblunt vector (Solgent, Korea). A DNA region ranging from the promoter to terminator was amplified from pSVAmalFXNt6H::*sso3032* and ligated between U and D using *Sac*II restriction and ligation. *pyrEF* gene was amplified from *S. solfataricus* P2 as a selection marker and ligated into the constructed vector through the BamHI restriction site. Finally, the constructed vector, pTBlunt::U-*sso3032*-D-*pyrEF*, was transformed to *S. acidocaldarius* MW001 via electroporation. The pop-in mutant was selected by the capacity of uracil biosynthesis. To pop out the vector DNA and *pyrEF* marker gene, the mutants were transferred to media supplemented with 10 mg/l of uracil and 100 mg/l of 5-fluoroorotic acid. In addition, 0.2% XOS was added to the selection plate to separate the wild-type and mutant growth rates (Fig. S1). For the construction of LAR1-1, which harbors both β-xylosidase and xylanase, pC::*sso1354* was transformed into LAR1.

### Protein Purification

Protein purification was conducted to characterize xylanase and β-xylosidase. As xylanase is anchored to the cell membrane [24], membrane fractions of MW001 and MW001/1354 were partially purified and their xylanase activities were compared. Cells were harvested when the OD reached 1.0 and resuspended to phosphate buffer (20 mM sodium phosphate with pH adjusted to 7.4, 500 mM NaCl, and 20 mM imidazole) and lysed by ultrasonication. The membrane fractions were separated from the lysates by centrifugation (20,000 ×g, 40 min, 4°C). The membrane-bound protein, collected as a pellet after centrifugation, was resuspended in 1 ml phosphate buffer. To purify the recombinant β-xylosidase, MW001/3032 was cultivated and harvested when the OD reached 1.0. Cells were lysed as described above, and the supernatants were filtered and further purified by Ni-NTA affinity chromatography. The bound proteins were washed with 50 mM imidazole in the same buffer (20 mM sodium phosphate pH 7.4, 0.5 M NaCl, and 50 mM imidazole) and eluted using 200 mM imidazole buffer (20 mM sodium phosphate pH 7.4, 0.5 M NaCl, and 200 mM imidazole). The protein concentration was determined by Bradford’s method using bovine serum albumin as a standard [28].

### Enzyme Characterization

The characterization of xylanase was conducted by following the method of Cannio *et al*. with slight modification [22]. First, 0.1% of Remazol Brilliant Blue R-D-xylan (RBB-xylan) was incubated with 10 μg of membrane fraction in 50 mM sodium acetate buffer (pH 4.0). Then, the reaction was conducted at 90°C for 30 min and inactivated by adding 400 μl of ethanol. After 15 min of resting, samples were centrifuged (16,000 ×g, 5 min, 4°C), and 200 μl of the supernatant was used to measure enzyme activity. Enzyme activity was detected colorimetrically at 590 nm, and RBB was used as a standard.

The characterization of β-xylosidase was performed with 4-nitrophenol-β-D-xylopyranoside (pNPX) as a substrate. The reaction was conducted at 90°C for 10 min in a final volume of 200 μl of the reaction mixture containing 50 mM sodium acetate buffer (pH 6.0), 1 mM pNPX, and 1 μg of the purified enzyme. The reaction was stopped by adding 100 μl of 1 M sodium carbonate. The released pNP was quantified colorimetrically at 420 nm, and pNP served as a standard.

For pH effect, the temperature of the reaction mixture containing xylanase and β-xylosidase was fixed at 90°C. Sodium citrate (pH 3.0–4.0), sodium acetate (pH 4.0–6.0), and sodium phosphate (pH 6.0–9.0) were selected as pH buffers. For temperature effect, xylanase and β-xylosidase were adjusted to pH 4.0 and 6.0, respectively, and enzyme activity was determined between 60 and 100°C.

### Sugar Uptake Experiment

Sugar uptake experiment was conducted by following the method of Choi *et al*., with minor modification [29]. Cells were grown in 50 ml of Brock’s media supplemented with 0.2% XOS and 0.02% dextrin, and the cells were harvested when the OD reached 0.5–0.6. The harvested cells were washed with Brock’s basal media three times and resuspended to 5 ml of 1% XOS dissolved in Brock’s basal media, followed by incubation at 77°C. After 0, 12, and 48 h of incubation, 300 μl of the samples were taken and the cell pellets and supernatants were separated by centrifugation. Then, the cell pellet was resuspended in 300 μl of Brock’s basal media and lysed by sonication, followed by centrifugation to remove cell debris. XOS transported into the cells was visualized by TLC following the method by Reiffová *et al*., with minor modification [30]. In detail, 7 μl of samples were loaded on silica gel 60 F_254_ plates, and *n*-butanol/acetic acid/water (20:10:2, v/v/v) was used as a mobile phase. Staining was conducted with methanol solution containing 0.12% (w/v) naphthol and 10% (v/v) sulfuric acid. The spots were visualized after being placed in an oven at 110°C for 7 min.

### Hydrolysis Pattern Analysis of Xylan and XOS

The hydrolysis pattern of xylan and XOS reacted with xylanase and β-xylosidase, and both enzymes were analyzed by TLC. For the β-xylosidase assay, the soluble protein (140 μg) of MW001 or purified β-xylosidase (6.85 μg) was incubated with hemicellulosic substrates. XOS or xylan was given at a final concentration of 1% in the reaction mixture containing 50 mM sodium acetate (pH 6.0). The enzyme reaction was conducted at 80°C overnight, and the hydrolyzed products were examined by TLC. For xylanase assay, 160 μg of membrane-bound enzymes were incubated with 1% xylan in 50 mM sodium acetate (pH 4.0) at 80°C overnight. The result of xylanase reaction was visualized by TLC.

For the synergetic action of xylanase and β-xylosidase, enzyme reactions were performed following two successive steps: first, xylan or XOS was incubated with 150 μg of membrane-bound fractions in 50 mM sodium acetate buffer (pH 4.0) at 80°C overnight, and then, 100 μl of the reaction mixture was transferred to the second mixture containing 50 mM sodium acetate buffer (pH 6.0) and 8 μg of purified β-xylosidase. This reaction continued at 80°C overnight. After the reaction, 1 μl of the reaction mixture was loaded on a silica gel TLC plate and visualized as described above.

## Results and Discussion

### Inability of *S. acidocaldarius* to Utilize XOS and Xylan

**Fig. 1:**
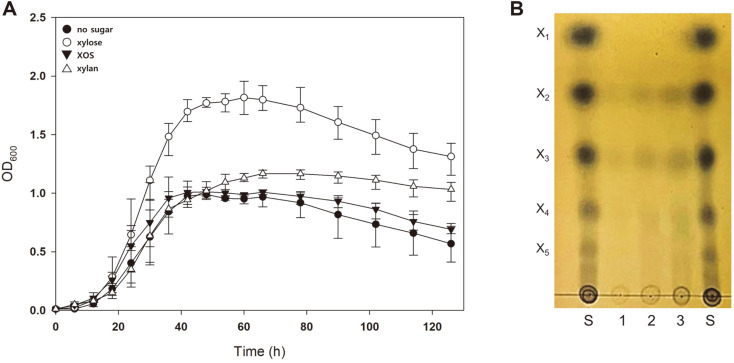
*S. acidocaldarius* MW001 cannot utilize XOS and xylan. (**A**) Growth of MW001 in the presence of hemicellulosic biomass. For the basal growth of MW001 strain, 0.2% NZ-amine and 0.02% dextrin were added to Brock’s medium. Various types of hemicellulosic biomass, including xylose (open-circle), XOS (closed-triangle), and xylan (opentriangle), were supplemented to the basal media at a concentration of 0.2% or no additive sugars were provided (closed-circle). MW001 strain was inoculated with an initial OD of 0.01, which was measured every 6 h. All experiments were conducted with triplicate and upper and lower side error bars representing maximum and minimum OD, respectively. (**B**) TLC chromatogram of XOS transported into *S. acidocaldarius* MW001. Cells were incubated with 1% XOS, and XOS transported into the cells was visualized by TLC. Xylose and XOS (X2–X5) were used as standards. S, sugar standard; 1, cell lysate after the 0 h incubation; 2, after 12 h; 3, after 48 h.

To confirm hemicellulosic biomass utilization by *S. acidocaldarius*, the wild-type strain (MW001) was grown in Brock’s media supplemented with 0.2% NZ-amine and 0.02% dextrin. In addition, 0.2% XOS or 0.2% xylan was added to the media to compare cell growth dependence on the presence of hemicellulosic biomass. MW001 growth using various carbon sources was detected at 6-h intervals. As shown in Fig. 1A, the OD of MW001 reached over 1.8 when xylose was provided as a carbon source. Meanwhile, when only 0.02% dextrin was given, the maximum growth of the cells increased slightly, reaching OD 1.0. This shows that this strain utilizes xylose well as a carbon source, which agrees with the results of previous studies [16, 20, 21]. In the case of MW001 grown in the presence of XOS and xylan, the MW001 strain had a maximum OD of 1.0, similar to the growth of MW001 in the absence of any carbon source other than 0.02% dextrin, suggesting that wild-type MW001 cannot utilize XOS or xylan for cell growth. Therefore, for *S. acidocaldarius* to utilize hemicellulosic biomass, it is necessary to introduce the heterologous genes responsible for the degradation of xylan and the uptake of the degradation products into the cells.

The uptake capacity for XOS was measured to determine the inability of the MW001 strain to uptake XOS into the cells, or uptake it into the cells but not metabolize it. TLC analysis of the cell lysate showed that the spots of XOS transported into *S. acidocaldarius* grown with XOS gradually increased, suggesting that *S. acidocaldarius* could uptake XOS into the cells (Fig. 1B). From the result of the uptake experiment, we speculated that *S. acidocaldarius* can uptake XOS, but cannot metabolize XOS by breaking it down to xylose.

### β-Xylosidase Expression Enables *S. acidocaldarius* to Utilize XOS

**Fig. 2:**
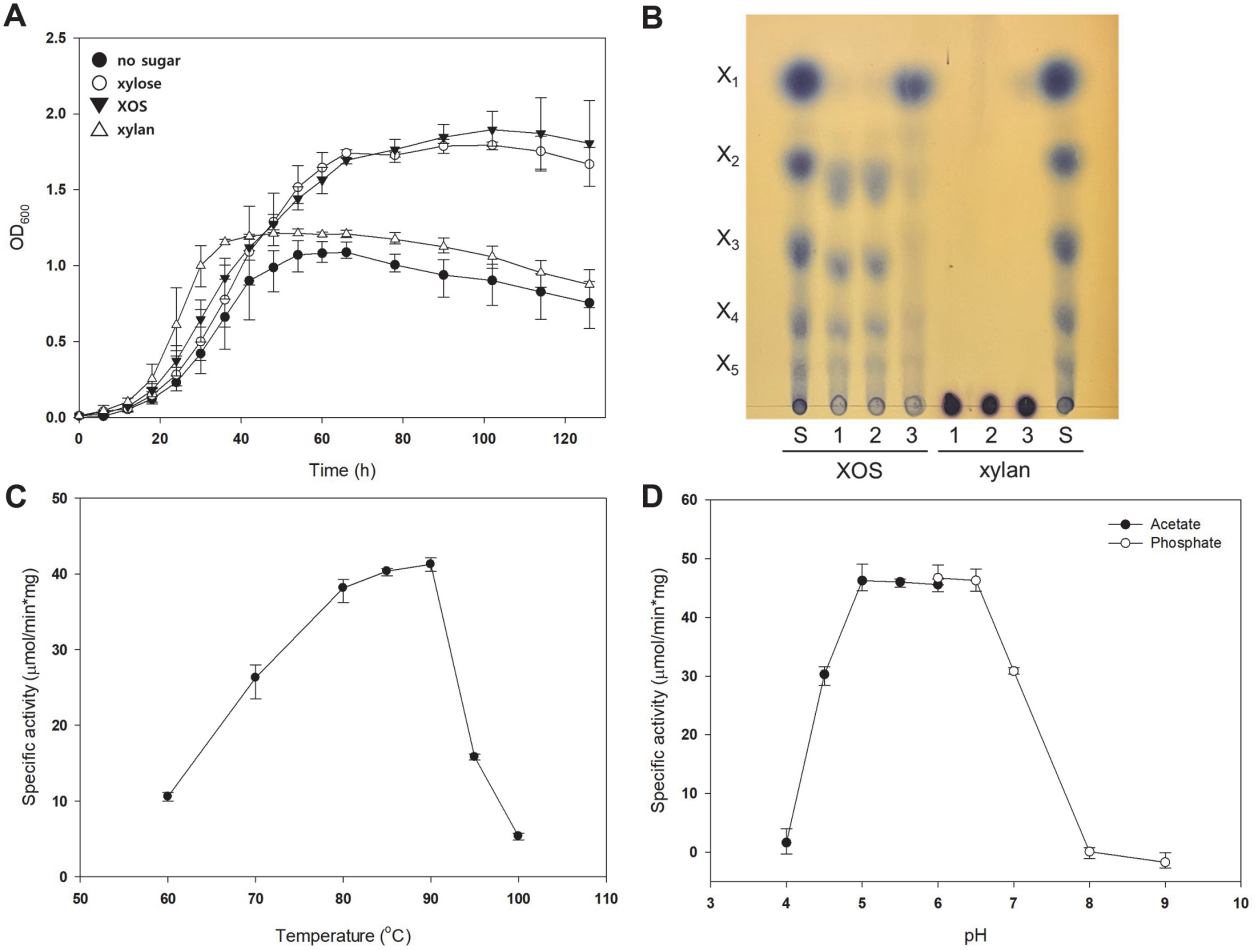
Expression of β-xylosidase enables *S. acidocaldarius* to utilize XOS. (**A**) Growth of MW001/3032 in the presence of XOS and xylan. MW001/3032 strain, containing β-xylosidase, was grown in Brock’s medium supplemented with 0.2% NZ-amine and 0.02% dextrin. To compare cell growth patterns when an additional carbon source was provided, xylose (open-circle), XOS (closed-triangle), and xylan (open-triangle) or no sugar (closed-circle) was supplemented in the culture medium at a concentration of 0.2%. The initial OD of MW001/3032 was 0.01, and cell growth was measured at 6-h intervals. Error bars represent the maximum and minimum values of triplicates. (**B**) TLC chromatogram of XOS and xylan hydrolysis by MW001/3032. The cell-free extracts from MW001 and MW001/3032 were incubated with 5% XOS or xylan, and the reaction products were visualized by TLC. Xylose and XOS (X2–X5) served as standards. S, sugar standard; 1, no enzyme treated; 2, MW001 treated; 3, MW001/3032 treated. (C and D) Temperature and pH profiles of recombinant β-xylosidase. The recombinant β-xylosidase was purified from MW001/3032. The enzymatic reaction was conducted with 1 mM pNPX and 1 μg of the enzyme solution for 10 min in 50 mM of each buffer and at various temperatures, as described in the Materials and Methods section.

Since *sso3032* from *S. solfataricus* was identified to encode β-xylosidase/α-arabinofuranosidase in previous studies [23, 31, 32], the gene was introduced into the *S. acidocaldarius* MW001 strain. MW001/pSVAmalFX-Nt6H::*sso3032* (MW001/3032) was grown in a medium supplemented with 0.2% xylose, XOS, xylan, or without sugar. For heterogenous β-xylosidase expression, 0.02% dextrin was additionally provided to each medium because the *mal* promoter of pSVAmalFX-Nt6H was induced in the presence of maltodextrin. As shown in Fig. 2A, the mutant grown with 0.2% XOS showed a growth pattern similar to that of a positive control, the mutant grown in Brock’s medium supplemented with 0.2% xylose, indicating that the introduction of β-xylosidase enables *S. acidocaldarius* to utilize XOS. Nevertheless, the mutant still could not grow sufficiently when xylan was introduced as a carbon source, suggesting that additional enzymes are needed for xylan utilization.

The optimal pH and temperature of the purified recombinant β-xylosidase were determined using pNPX as a substrate. As shown in Figs. 2C and 2D, purified β-xylosidase showed maximum activity at 90°C and maintained the activity at a pH of 5.0–6.5. In a study by Morana *et al*. [23], β-xylosidase expressed in *E. coli* showed maximum activity at 80–85°C and pH 6.5. Although the pH range in which β-xylosidase showed such maximum activity was similar, the optimal temperature of β-xylosidase expressed in *S. acidocaldarius* was higher than that of the enzyme expressed in *E. coli*. The difference in optimal temperature conditions between the enzymes expressed in *S. acidocaldarius* and *E. coli* appears to be a difference in the expression host. Sulfolobales, including *S. acidocaldarius*, *S. solfataricus*, and *S. shibatae*, are known to have post-translational modification systems, such as glycosylation, phosphorylation, methylation, disulfide bonds, and acetylation, and these modifications may contribute to the thermostability of proteins [33, 34].

The hydrolysis pattern of XOS and xylan by MW001/3032 was compared with that of MW001. The cell-free lysates of MW001 and MW001/3032 were incubated with XOS and xylan, and the reaction products were examined by TLC (Fig. 2B). The cell-free lysates of MW001/3032 were seen to have decomposed XOS into xylose, but those of MW001 did not decompose XOS, suggesting that β-xylosidase was expressed in MW001/3032 degraded XOS into xylose. When xylan was used as a substrate, none of the cell-free lysates degraded xylan, indicating that β-xylosidase alone cannot degrade xylan. This shows that MW001/3032 grew well with XOS but not in the medium supplemented with xylan.

### Expression of Xylanase Alone Does Not Enable *S. acidocaldarius* to Utilize Xylan

**Fig. 3:**
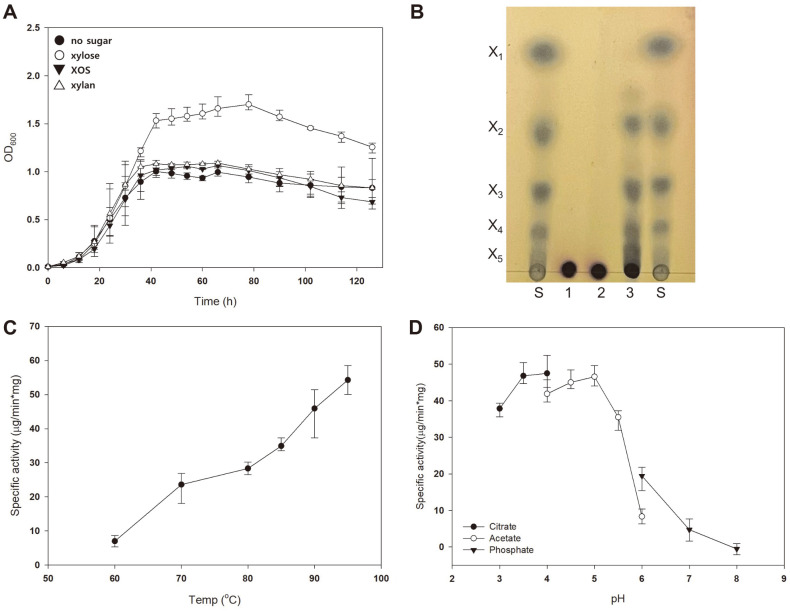
Expression of xylanase alone does not enable *S. acidocaldarius* to utilize xylan. (**A**) Growth of MW001/ 1354 in the presence of XOS and xylan. The growth pattern of MW001/1354 grown without sugar supplementation (closedcircle) or with supplementation of sugars, such as xylose (open-circle), XOS (closed-triangle), and xylan (open-triangle), was compared. Brock’s medium was supplemented with 0.2% NZ-amine and 0.02% dextrin. Initial inoculation of MW001/1354 was conducted at an OD of 0.01, and cell growth was measured at 6-h intervals. Experiments were conducted in triplicates, and error bars showed the maximum and minimum OD. (**B**) TLC chromatogram of xylan hydrolysis by MW001/1354. The cellfree extracts from MW001 and MW001/1354 were incubated with 5% xylan, and the reaction products were visualized by TLC. Xylose and XOS (X2–X5) served as standards. S, sugar standard; 1, no enzyme treated; 2, MW001 treated; 3, MW001/1354 treated. (C and D) Temperature and pH profiles of recombinant xylanase. The recombinant xylanase was partially purified from MW001/1354. The enzyme reaction was conducted with 0.1% RBB-xylan and 10 μg of the enzyme solution for 30 min in 50 mM of each buffer and at various temperatures, as described in the Materials and Methods section.

To make the MW001 strain utilize xylan, *sso1354*, which encodes protein harboring cellulase and xylanase activity, was introduced into *S. acidocaldarius* MW001. The growth pattern of MW001/1354 in the presence of various sugars was compared (Fig. 3A). MW001/1354 grew efficiently when xylose was provided as a carbon source. However, the maximum OD of the mutant grown in Brock’s medium supplemented with XOS or xylan was similar to that of the mutant exposed to 0.02% dextrin, suggesting that the solo action of xylanase cannot make *S. acidocaldarius* utilize xylan or XOS. The pH and temperature profiles of the recombinant xylanase were examined and compared to the xylanase previously reported by Maurelli *et al*. [24]. The recombinant xylanase partially purified from MW001/1354 showed maximum activity at pH 3.5–4.0 and a temperature of 95°C (Figs. 3C and 3D). These properties correspond well with the results of the xylanase extracted from *S. solfataricus* Oα, which showed maximum activity at pH 4.0 and a temperature of 90°C.

When the membrane-bound fraction of MW001/1354 was incubated with xylan, the xylan was degraded into XOS (Fig. 3B), thus suggesting that the heterologously expressed xylanase can degrade xylan effectively. However, spots X2–X5 appeared after the enzyme reaction, indicating that xylanase can hydrolyze xylan into XOS, but not xylose. Since the mutant cannot hydrolyze XOS into xylose due to the lack of β-xylosidase, this strain was unable to utilize xylan or XOS for its growth. This result suggests that the cooperative action of xylanase and β-xylosidase in utilizing xylan as a carbon source in *S. acidocaldarius* is necessary.

### Synergistic Activity of Xylanase and β-Xxylosidase in *S. acidocaldarius* Is Required to Utilize Xylan

**Fig. 4:**
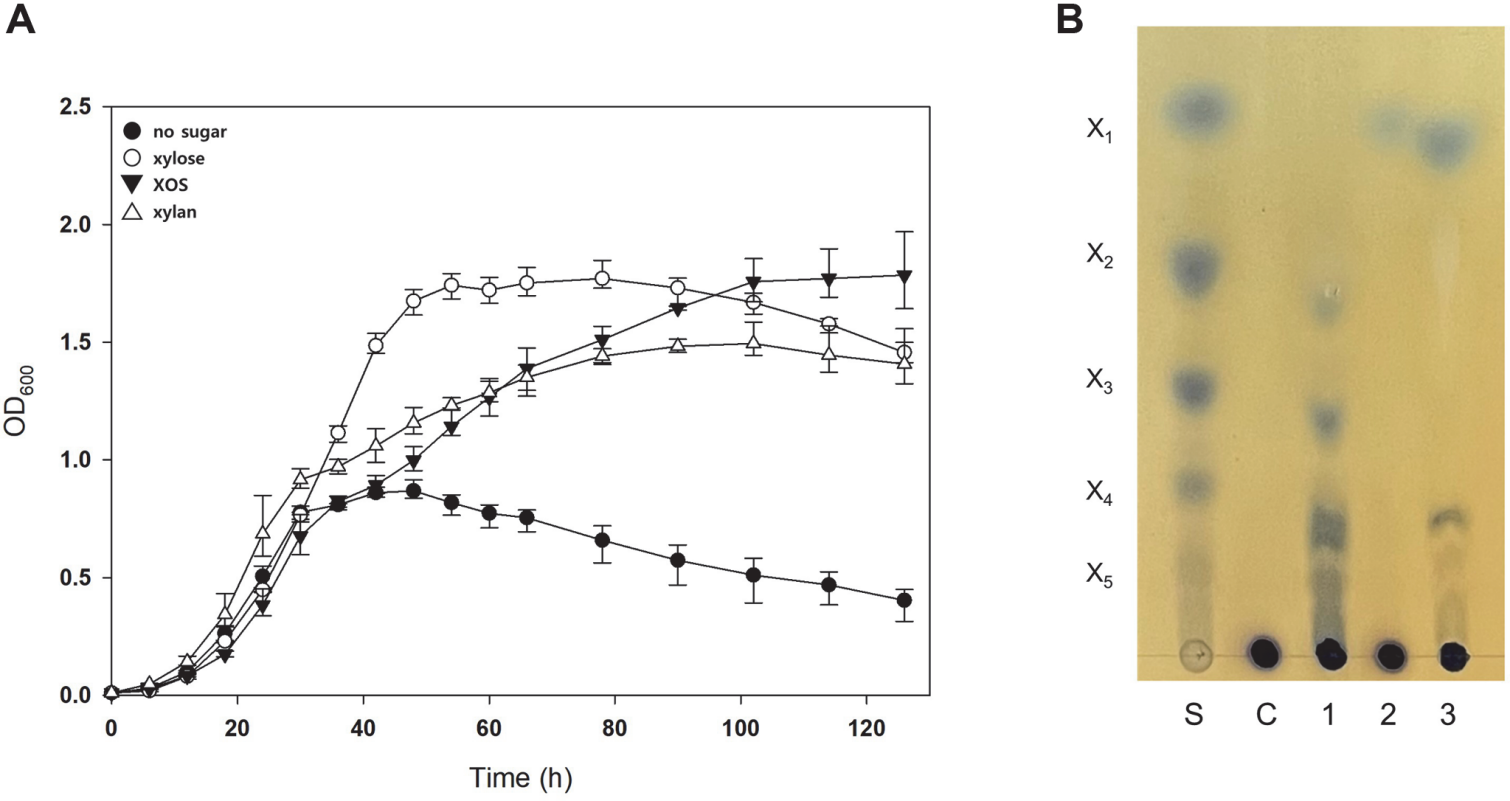
Synergistic activity of xylanase and β-xylosidase in *S. acidocaldarius* is required to utilize xylan. (**A**) Growth of LAR1-1 in the presence of hemicellulosic sugars. Each medium contains 0.2% NZ-amine and 0.02% dextrin. Cell growth in 0.2% xylose (open-circle), XOS (closed-triangle), xylan (open-triangle), or without sugar (closed-circle) was compared. Error bars represent the maximum and minimum OD values of triplicate experiment, respectively. (**B**) The synergetic action of β-xylosidase and xylanase toward hemicellulosic biomass. The membrane fraction of MW001/1354 and cell-free extracts from MW001/3032 were incubated with 5% XOS or xylan, and the reaction products were visualized by TLC. Lane S, xylose and XOS (X2–X5) standard; Lane C, no enzyme treated; Lane 1, MW001/1354 treated; Lane 2, MW001/3032 treated; Lane 3, LAR1-1 treated.

To introduce two individual genes (*sso3032* and *sso1354*) into *S. acidocaldarius* MW001, *sso3032* was ligated into genomic DNA by the markerless insertion method. Then, the constructed mutant, *LAR1*, was further mutated by introducing *sso1354* via the transformation of the protein expression vector. The constructed mutant LAR1-1 strain containing *sso3032* and *sso1354* was effectively grown in Brock’s medium supplemented with 0.2%xylan, further demonstrating that the synergistic activities of xylanase and β-xylosidase enable *S. acidocaldarius* to utilize xylan (Fig. 4A).

A two-step reaction was conducted with xylan to confirm the cooperative action of xylanase and β-xylosidase, and the reaction of β-xylosidase rarely broke down xylan. While xylanase can convert xylan into XOS, this enzyme cannot hydrolyze XOS into xylose, a sugar that *S. acidocaldarius* can use as a carbon source (Fig. 4B). When xylan was reacted with xylanase and further with xylosidase, xylose was produced by the hydrolysis of two enzymes; therefore, the cells can grow efficiently in the presence of xylan. This result suggests that the synergistic action of xylanase and β-xylosidase is essential for the degradation and utilization of xylan in *S. acidocaldarius*.

### Additional Enzymatic Reaction Is Needed for Engineered *S. acidocaldarius* to Utilize Cellulosic Biomass

**Fig. 5:**
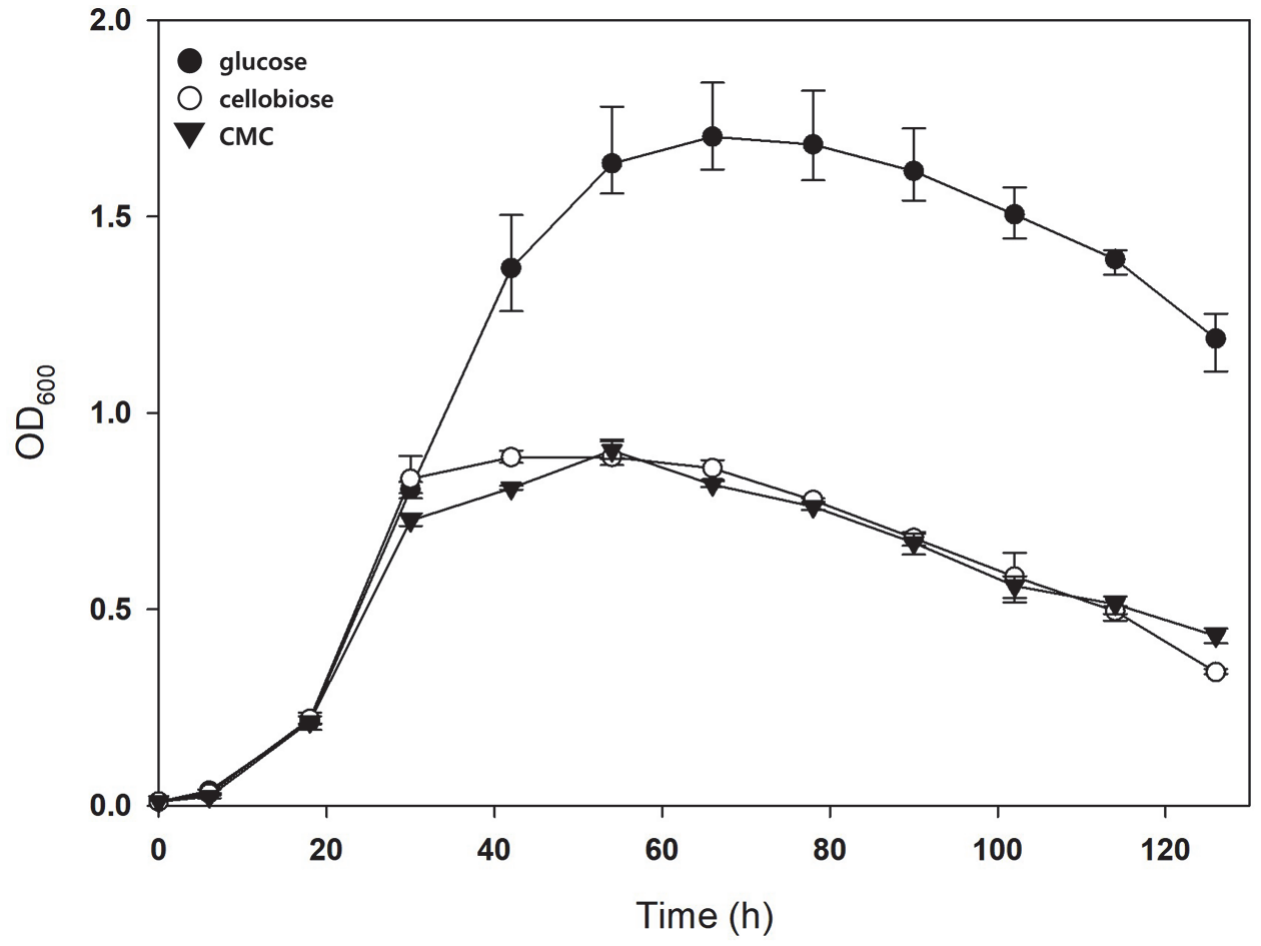
Growth of LAR1-1 in the presence of cellulosic biomass. Each medium contains 0.2% NZ-amine and 0.02% dextrin. Cell growth in 0.2% glucose (closed-circle), COS (open-triangle), and CMC (closed-triangle) was compared. Error bars represent the maximum and minimum OD values of triplicate experiment, respectively.

Since SSO1354, which harbors xylanase activity, is also known to possess endoglucanase activity [24, 35], we speculated that LAR1-1 may degrade cellulosic polymer into cellooligosaccharide (COS). To see whether the LAR1-1 mutant can utilize cellulosic sugar, the mutant was grown in Brock’s medium supplemented with 0.2%cellobiose or CMC. Unlike the result of the mutant grown in XOS or xylan, the growth pattern with cellobiose or CMC was similar to the cells grown without sugar supplementation (Fig. 5). As SSO1354 was identified to harbor both xylanase and cellulase activities, the absence of an enzyme that can convert COS into glucose seems to be responsible for this result. β-glucosidase, which is encoded by lacS, is inactive in *S. acidocaldarius*, unlike the β-glucosidase of *S. solfataricus* [18, 19]. Thus, the LAR1-1 strain’s inability to utilize cellulosic biomass can be overcome by the expression of active β-glucosidase.

In this study, we aimed to develop a strain that can utilize hemicellulosic biomass as a carbon source. The synergetic action of two recombinant enzymes, xylanase and β-xylosidase of *S. solfataricus*, enabled *S. acidocaldarius* to utilize xylan and XOS. Thus, for the simultaneous utilization of cellulosic and hemicellulosic biomasses, additional enzymes that can convert COS into glucose need to be expressed in the LAR1-1 strain.

## Supplemental Materials

Supplementary data for this paper are available on-line only at http://jmb.or.kr.

## Figures and Tables

**Table 1:** Strains and plasmids used in this study.

Strains or plasmids	Genetic marker and characteristics	Reference
Strains		
*S. acidocaldarius* DSM639	*S. acidocaldarius* wild-type strain	[36]
MW001	DSM639 derivative, uracil auxotrophic mutant, Δ*pyrE*	[18]
MW001/3032	MW001 derivative, containing pSVAmalFX-Nt6H::*sso3032*	In this study
MW001/1354	MW001 derivative, containing pC::*sso1354*	In this study
LAR1	MW001 derivative, replacement of *pyrE* and *pyrF* (*saci_1597* and *saci_1598*) gene with *sso3032*	In this study
LAR1-1	*LAR1* derivative, containing pC::*sso1354*	In this study
Plasmids		
pC	*E. coli*-*Sulfolobus* shuttle vector, Amp^r^	[37]
pSVAmalFX-Nt6H	*E. coli*-*Sulflobus* shuttle vector, containing P*_saci1165_* and C-terminal His_6_ tag	[25]
pC::*sso1354*	For expression of SSO1354, pC derivative, containing P*_gdhA_* fused with *sso1354* gene	In this study
pSVAmalFX-Nt6H::*sso3032*	For expression of SSO3032, pSVAmalFX-Nt6H derivative, containing *sso3032* gene	In this study
pTB::U-*sso3032*-D-*pyrEF*	For the construction of *LAR1* strain, pTblunt derivative	In this study

**Table 2:** Primers used in this study.

Primer	Sequence (5'→3')	Remarks
NE 05	AGTCCGCGGTTCTCCACTGTTTACGTT	For expression of *sso1354*, specific for P*_gdhA_*, containing *Sac*II restriction site
NE 13	ATATAATTTATTCATCGCAGAAGAATTCATATT	For expression of *sso1354*, specific for P*_gdhA_*, introducing upstream region of *sso1354*
NE 14	AATTCTTCTGCGATGAATAAATTATATATTGTG	For expression of *sso1354*, specific for *sso1354*, introducing downstream region of P*_gdhA_*
NE 15	GAGGAGAGTTTCAGAAAAGTTGGATAC	For amplification of *sso1354*
NE 16	TTA*ATGATGATGATGATGAT*GGAGGAGAGTTTCA	For expression of *sso1354*, specific for downstream of *sso1354*, introducing 6x His-tag and stop codon
NE 17	*CATCATCATCATCATCAT*TAAACAATATAAGAC	For expression of *sso1354*, specific for terminator region of *sso1354*, introducing 6x His-tag and stop codon
NE 18	ATCCGCGGATGCTTACACTACCTACGATG	For expression of *sso1354*, specific for terminator region of *sso1354*, containing *Sac*II restriction site
AR 105	ATGGATTTCGTGAAAGCTCTAC	For markerless insertion of *sso3032*, specific for *pyrE* (*saci_1597*)
AR 106	GTTTTTCCCGCGGCTTTAAGAATTGAACCACC	For markerless insertion of *sso3032*, specific for *pyrE* (*saci_1597*), introducing *Sac*II and upstream region of *pyrF* (*saci_1598*)
AR 107	CTTAAAGCCGCGGGAAAAACTATCTTGACAG	For markerless insertion of *sso3032*, specific for *pyrF* (*saci_1598*), introducing *Sac*II and downstream region of *pyrE* (*saci_1597*)
AR 108	TCATGTTTGCCGAACTTTAC	For markerless insertion of *sso3032*, specific for *pyrF* (*saci_1598*)
AR 109	CCGCGGCCAGATATCTGATAGTTGG	For markerless insertion of *sso3032*, specific for P_mal_ in pSVAmalFX-Nt6H, containing *Sac*II restriction site
AR 110	CCGCGGTCAATGGTGATGATGGTGATG	For markerless insertion of *sso3032*, specific for downstream region of *sso3032* in pSVAmalFX-Nt6H, containing *Sac*II restriction site
KH 71	GGATCCAATGAAACTACTTTCCCTGATAGATAA	For markerless insertion of *sso3032*, specific for *pyrE* (*sso0615*), containing BamHI restriction site
AR 111	GGATCCTTATAAAGACCGGCTATTTTTTCAC	For markerless insertion of *sso3032*, specific for *pyrF* (*sso0616*), containing BamHI restriction site
AR 180	ATATATGCTCTTCTAGTACAGCTATAAAGAGTCT CCTAAATCAG	For expression of *sso3032*, containing *Sap*I restriction site
AR 181	TATATAGCTCTTCATGCCTCTATTTGTACATTTG ATAGAAATAT	For expression of *sso3032*, containing *Sap*I restriction site

Restriction sites are underlined.
