# Supplementary material for: Engineering of Sulfolobus acidocaldarius for Hemicellulosic Biomass Utilization
Source: J Microbiol Biotechnol. 2022 Feb 24;32(5):663–71. doi: 10.4014/jmb.2202.02016 (PMC9628888; doi:10.4014/jmb.2202.02016)
Supplement: Supplementary file 1 [file jmb-32-5-663-supple.pdf]

J. Microbiol. Biotechnol.
https://doi.org/10.4014/jmb.2202.02016

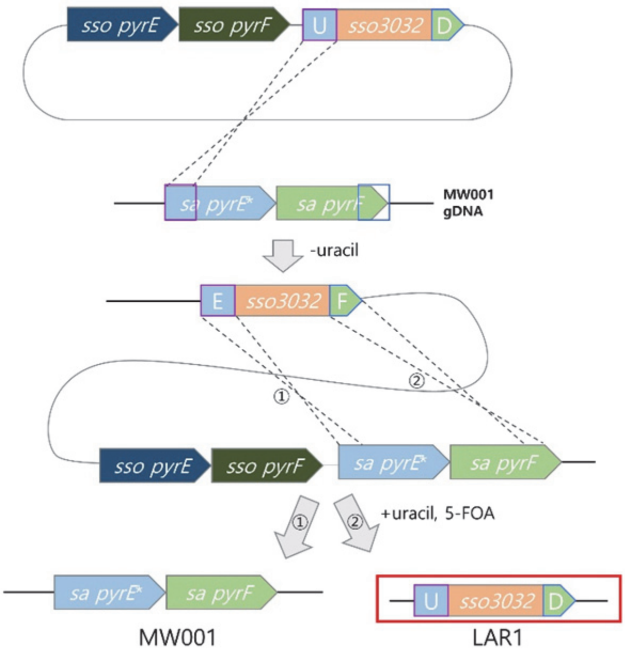

Figure S1. A scheme of LAR1 construction using markerless insertion. A plasmid harboring U–*sso3032*–D and *pyrE–pyrF* amplified from *S. solfataricus* was introduced to *S. acidocaldarius* MW001, which has inactivated *pyrE* gene. After the transformation, a strain who proceeded homologous recombination on U (or D) locus can survive under the uracil deficient condition. After the selection of the mutant (pop-in mutant) who introduced plasmid DNA into genomic DNA on uracil deficient plate, the selected pop-in mutant was transferred to the media supplemented with uracil and 5-FOA. As the cell with active *pyrE* gene cannot survive under the presence of 5-FOA, only the mutant who discard *pyrE* gene of *S. solfataricus* from the genomic DNA by homologous recombination can survive. Two types of strain can be detected by the selection with 5-FOA, and the constructed strain can be identified by sequencing. Dashed lines represent the locus where homologous recombination proceeded.
